# An Evaluation of the Antibacterial Activity of *Pterocarpus tinctorius* Bark Extract against Enteric Bacteria That Cause Gastroenteritis

**DOI:** 10.1155/2022/7973942

**Published:** 2022-09-27

**Authors:** Isaac Mphande, Andrew Kataba, Kaampwe Muzandu, Angela Gono-Bwalya

**Affiliations:** ^1^Department of Pharmacy, School of Health Sciences, University of Zambia, Lusaka, Zambia; ^2^Department of Biomedical Sciences, School of Veterinary Medicine, University of Zambia, Lusaka, Zambia

## Abstract

Enteric bacteria are the leading cause of bacterial gastroenteritis worldwide, particularly in low-income countries. The bark decoction of *Pterocarpus tinctorius* (Fabaceae) has traditionally been used to treat bacterial gastroenteritis. However, studies reporting the antibacterial activity of *Pterocarpus tinctorius* are rare. Therefore, this study aimed to evaluate the antibacterial activity of stem bark extract of *Pterocarpus tinctorius* against *Escherichia coli*, *Salmonella typhi*, and *Shigella dysenteriae*. The powdered bark extract was successively extracted with methanol using the cold continuous maceration method, followed by partitioning the crude methanolic extract to obtain methanolic, hexane, and chloroform subextracts. Three fractions were isolated from the methanolic subextract using ordinary normal phase column chromatography. The antibacterial activity of the extracts and fractions was performed using the agar well diffusion method. The minimum inhibitory concentration (MIC) was determined using the agar well diffusion method. While, minimum bactericidal concentration (MBC) was obtained by the subculturing method. The methanolic subextract was the only extract that showed antibacterial activity against the tested bacteria, and its activity was highest on *Shigella dysenteriae* followed by *Salmonella typhi* and was least active on *Escherichia coli*, with mean inhibition zones of 14.3 ± 0.2, 13.7 ± 0.3, and 12.2 ± 0.1 at 200 mg/mL, respectively. Chloroform subextract showed antibacterial activity only on *Shigella dysenteriae*, while hexane subextract did not show antibacterial activity against all bacteria tested at 100 mg/mL and 200 mg/mL. Among the three subfractions of methanolic subextract, only one subfraction was active and had both mean minimum inhibitory concentration and a minimum bactericidal concentration against *Escherichia coli* at 1.25 mg/mL, *Salmonella typhi* at 1.25 mg/mL, and *Shigella dysenteriae* at 0.6 mg/mL. The findings of this study support the use of *Pterocarpus tinctorius* in traditional medicine. Therefore, purification and structural elucidation studies are highly recommended.

## 1. Introduction

Bacterial gastroenteritis is a major public health concern worldwide [1]. Globally, there are nearly 1.7 billion cases of childhood gastroenteritis every year [2]. However, enteric bacteria such as *Salmonella* and *Shigella* species are among the leading causes of bacterial gastroenteritis worldwide [3–5]. Literature also confirms that bacterial gastroenteritis in low-income countries is mainly caused by certain *Salmonella*, *Escherichia coli,* and *Shigella* species [6, 7]. These bacteria have been listed by the World Health Organization as priority pathogens to be considered when conducting research and developing new and effective antibacterial treatments [8]. Although synthetic drugs have replaced plants as the source of most medicinal agents in high-income countries, plants produce a variety of bioactive secondary metabolites which are used to treat chronic and infectious diseases [9]. In low-income countries, people living in remote rural or tribal areas use indigenous plants to treat gastroenteritis as the knowledge reaches them through the experiences of parental generations [10], which they consider to be part of their culture and heritage [9]. Moreover, synthetic antibiotics such as fluoroquinolones and quinolones cause long-lasting, disabling, and potentially permanent serious side effects [11]. However, plants have potent antibacterial bioactive compounds [12], with fewer side effects [9]. This causes some people, even in high-income countries, to use plants as alternative medicines to treat infectious diseases [9]. Most medicinal plants belong to the Fabaceae family, which is most commonly used in the treatment of various conditions and diseases [13, 14]. One genus belonging to the Fabaceae family is *Pterocarpus*. The genus *Pterocarpus* consists of about 30 species found in the tropics, except in Australia and Madagascar [15]. In Africa, Asia, and Latin America, some members of the genus are used in ethnomedicine to treat diseases and conditions such as inflammation, pain, cardiovascular, gastrointestinal, diarrhoea, headache, stomachache, schistosomiasis, sores, gonorrhoea, ulcers, eye diseases, inducing vomiting, antihyperglycemic activity, antipyretic, anthelmintic, tonic, haemorrhage, dysentery, aphrodisiac, diaphoretic activities, mental aberrations, cooling agent, wound dressing, and skin diseases [16, 17]. The genus *Pterocarpus* is rich in polyphenolics [18], which have been reported to be effective against enteric bacteria [19, 20]. Qualitative phytochemical analysis of the heartwood of *Pterocarpus tinctorius* revealed the presence of phenols, flavonoids, and stilbenoids [21]. *Pterocarpus tinctorius* has several valuable antibacterial and medicinal properties [22]. However, there are limited scientific data reporting the antibacterial activity of *Pterocarpus tinctorius*. The aqueous extract of *Pterocarpus tinctorius* is used in ethnomedicine to treat bacterial gastroenteritis in the Chinsali district, Muchinga Province, Zambia. However, there are rare studies reporting antibacterial activity of *Pterocarpus tinctorius* against *Shigella*, *Salmonella,* and *Escherichia coli*. Therefore, this could be the first study to evaluate the antibacterial activity potential of the stem bark extracts of *Pterocarpus tinctorius* against enteric bacteria that cause bacterial gastroenteritis. This study cemented WHO guidelines which stipulate that *Shigella dysenteriae, Salmonella typhi*, and *Escherichia coli* should be bacteria of priority when researching the antibacterial activity of plants [8]. This is because these bacteria have acquired antimicrobial resistance in many parts of the world [8]. Therefore, this study is significant since it has provided scientific knowledge on the antibacterial activity of *Pterocarpus tinctorius* bark extracts against *Shigella dysenteriae*, *Salmonella typhi*, and *Escherichia coli*. It has also provided information on the classes of phytochemicals found in the bark of *Pterocarpus tinctorius,* which could be responsible for the antibacterial activity. Furthermore, the results are expected to stimulate further research regarding the antibacterial activity of *Pterocarpus tinctorius*.

## 2. Materials and Methods

### 2.1. Plant Material

The fresh bark of *Pterocarpus tinctorius* was collected from Mulakupikwa village, Chinsali district of Northern Zambia (east 413636, north 8825840). A plant specimen was deposited, identified, and authenticated at the University of Zambia, School of Natural Sciences, in the Department of Biological Sciences by a taxonomist.

### 2.2. Preparation of the Extract

The collected fresh bark of *Pterocarpus tinctorius* was dried in the open air under shade to prevent direct sunlight from inactivating the chemical constituents. Following drying, bark samples were pulverised into powder using a mechanical grinder. The 100 g of *P. tinctorius* powdered bark was weighed using a sensitive digital weighing balance (Adam Nimbus Group, Stuttgart, and Baden-Württemberg, Germany). The powder was macerated in 700 mL of analytical grade (99.9%) methanol (Sasol, Sandton, South Africa) in a 1 L beaker on a magnetic stirrer for 24 hours. After 24 hours of stirring, the extract was separated from the marc using gauze, and the resulting liquid was suction filtered through a Whatman No.1 filter paper using a Buchner funnel. The residue was remacerated, and the procedure was repeated three times to exhaustively extract the compounds from the plant material. The filtrates obtained from the successive maceration were dried under reduced pressure using a rotary evaporator at 40°C. The dried crude methanolic extract was then left in the desiccator for 24 hours to dry into a powder. Dried crude methanolic extracts (brown) were put in a labeled glass bottle and stored in the refrigerator at 4°C until use [23, 24].

### 2.3. Separation of Hexane, Chloroform, and Methanol Subextracts

To separate hexane subextract from methanolic crude extract, 100 mL of hexane was added to the separating funnel containing 5.1 g of the methanolic crude extract dissolved in 100 mL of methanol. The mixture in the separating funnel was well shaken while releasing pressure. It was then allowed to stand until two clear layers were formed. The lower layer of methanolic extract (brown) was then collected in the flat-bottomed flask through draining. The hexane layer (upper one, yellow), which remained in the separating funnel, was also drained from the separating funnel into the flat-bottomed flask. This process was repeated three times to exhaustively extract nonpolar compounds by hexane from the methanolic crude extract. Chloroform subextract was extracted from the methanolic extract (brown) that remained after extracting hexane subextract from the methanolic crude extract. This was achieved by adding 70 mL of chloroform to the separating funnel containing 100 mL of methanolic extract (brown). The mixture was well shaken while releasing pressure and allowed to stand until two clear layers were formed. The chloroform subextract (lower one) was drained from the separating funnel. This process was repeated three times to allow all the moderate polar compounds to be extracted by chloroform. After separating the chloroform subextract, what remained in the separating funnel was the methanolic subextract (M1). The three subextracts were evaporated at 40^o^C using a rotary evaporator and stored in the freezer at 4°C until use.

### 2.4. Separation of Subfractions Using Column Chromatography

**Figure 1:**
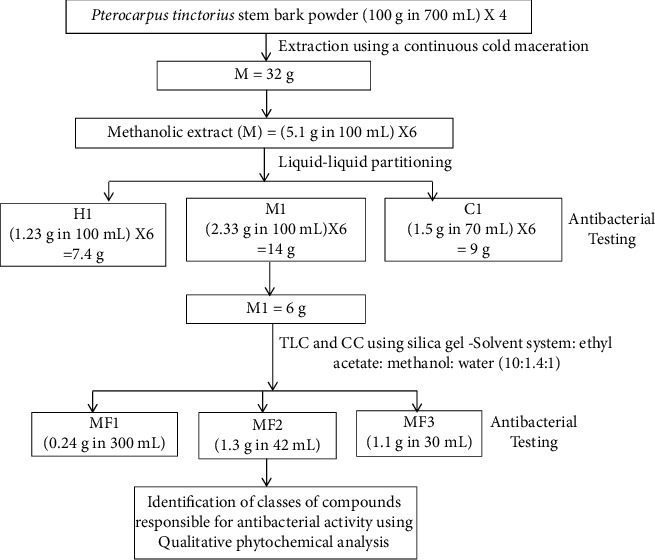
Representation of continuous procedure of extraction and fractionation of *Pterocarpus tinctorius*. FM1, least polar subfraction of methanolic subextract; FM2, medium polar subfraction of methanolic subextract; FM3, most polar subfraction of methanolic subextract; (M) methanolic extract M1, methanolic subextract; H1, hexane subextract; C1, chloroform subextract; TLC, thin layer chromatography; CC, column chromatography; MIC, minimum inhibitory concentration; MBC, minimum bactericidal concentration.

Then, isocratic column chromatography was performed as described by [25, 26] to separate the methanolic subextract (M1) into subfractions. Silica gel 80–120 mesh (HiMedia, Mumbai, Maharashtra, India) was used as the stationary phase (absorbent), while the mobile phase (eluent) used was ethyl acetate :  methanol :  water (10 : 1.4 : 1). 20 grams of silica gel were packed in a glass column for 1 gram of methanolic subextract. The isocratic method of elution was performed using a solvent system containing ethyl acetate, methanol, and water in the ratio of 10 : 1.4 : 1, respectively. Briefly, 6 grams of the methanolic subextract (M1) were dissolved in the minimum methanol solvent and added to a column packed with silica gel. The mobile phase ethyl acetate :  methanol :  water (10 : 1.4 : 1) was continuously poured onto the top of the column. The bottom outlet of the column was opened, allowing the eluent to flow through the column. As the eluent passed down the column, the compound fraction moved down the column. The separated fraction that flowed out of the column was collected in separate tubes. The fractions were evaluated using thin layer chromatography and the fractions having the same retardation factor (R_F_) values were combined to come up with subfractions FM1, FM2, and FM3. The three subfractions were dried using a rotary evaporator at 40°C and stored in the refrigerator at 4°C until use. A representation of the continuous procedure of extraction and fractionation of *Pterocarpus tinctorius* is shown in Figure 1.

### 2.5. Antibacterial Screening of Subextracts

The antibacterial activity of hexane, chloroform, and methanolic subextracts was determined using the agar well diffusion method described by [27]. Approximately 9 mL of Muller–Hinton agar (Oxoid, UK) was poured into sterile Petri plates (9 cm in diameter) and inoculated with the respective test organisms. Then, wells (6 mm) were punched out of the solid agar using sterile pipettes. Wells were filled with 50 uL of the hexane, chloroform, and methanolic subextracts (M1) at 200 mg/mL and 100 mg/mL. To validate the results, standard antibiotic disks of nalidixic acid (5 *µ*g/disk) and ciprofloxacin (5 *µ*g/disk) were used as positive controls on all the plates, while methanol, hexane, and distilled water were used as negative controls on the respective plates. The plates were kept at room temperature for 30 minutes to allow diffusion of the extract before incubation. The plates were incubated at 37°C for 24 hours. The antibacterial activity was assessed by measuring the diameter of the zone of inhibition formed around the well. The experiment was carried out in triplicates, and data were represented as the mean of three replicates ± standard deviation for each bacteria.

### 2.6. Antibacterial Screening of Subfractions

Three subfractions FM1, FM2, and FM3 isolated from the submethanolic extract using column chromatography were screened using the agar well diffusion method, as described by [28]. The samples from the fractions were reconstituted in 25% acetone, and all the fractions were tested for antibacterial activity at 10 mg/mL. An antibiotic (ciprofloxacin) at 0.01 mg/mL was used as a positive control, while 25% acetone was used as a negative control. The plates were kept at room temperature for 30 minutes to allow diffusion of the extract before incubation at 37°C for 24 hours. The antibacterial activity was assessed by measuring the diameter of the zone of inhibition formed around the well. The experiment was carried out in triplicates, and the data were represented as the mean of three replicates ± standard deviation for each type of bacteria.

### 2.7. Minimum Inhibitory Concentration (MIC)

The agar well diffusion technique with minor modification, as described by [29], was used to determine the MIC of the extract. A two-fold serial dilution was prepared in 25% acetone and diluted to achieve a decreasing concentration of 2.5 mg/mL, 1.25 mg/mL, 0.6 mg/mL, and 0.3 mg/mL, respectively. Ciprofloxacin at 0.01 mg/mL was used as the positive control. The MIC values were determined in triplicates. A 100 *μ*L of each dilution was introduced into Muller–Hinton agar plates already seeded with standardised inoculums (approximately 105 cfu mL^−1^) of *Shigella dysenteriae*, *Salmonella typhi*, and *Escherichia coli*. Acetone at 25% was used as a negative control. All test plates were incubated at 37°C for 24 hours. The least concentration of extract showing a clear zone of inhibition was regarded as the MIC.

### 2.8. Minimum Bactericidal Concentration (MBC)

The method and procedure for MBC were adopted from [30]. The concentrations obtained from the MIC experiment, ranging from 0.62 mg/mL to 2.5 mg/mL, which showed activity for the FM2 fraction, were used to determine MBC. Three test tubes were prepared for each of the concentrations. Test tubes containing 3 mL of 2.5 mg/mL, 1.25 mg/mL, and 0.62 mg/mL were reconstituted in one hundred microliters of sterile nutrient broth agar. One drop (0.025 mL) of the standardised bacteria was then suspended in each of the test tubes. The positive control was included in the assay to confirm antimicrobial susceptibility and was comprised of ciprofloxacin (0.005 mg/mL). The two negative controls used were solvent control and culture control. Solvent control contained only acetone at 25%, bacteria, and growth media, and its purpose was to find out if the solvent affected the bacteria growth. Culture control only contained bacteria and growth media, and its use was to find out whether the media could support microbial growth. The test tubes were then incubated at 37°C for 24 hours. Then, visual turbidity of the tubes was noted, both before and after incubation. And we compared the turbidity of the test tubes after incubation to the McFarland standard of 0.5 to confirm the growth of bacteria in the test tubes. Thereafter, a loopful from each of the tubes was streaked and subcultured on the sterile Mueller–Hinton agar and incubated for 24 hours at 37°C to determine the MBC. The concentration of the extract showing a bactericidal effect after 24 hours of incubation at 37°C was regarded as the MBC.

### 2.9. Phytochemical Analysis of Active Fractions

To determine the class of compounds responsible for the antibacterial activity of subfraction FM2, standard qualitative procedures as described by [31, 32] were used with some minor modifications. Phytochemical analysis of methanolic subfraction FM2 was conducted on saponins, tannins, anthraquinones, alkaloids, phenolics, terpenoids, and flavonoids as they have been reported to possess antibacterial activity against *Salmonella typhi*, *Shigella dysenteriae,* and *Escherichia coli* in the genus *Pterocarpus* [19, 27, 33].

### 2.10. Data Analysis

Data were organised and presented using tables and graphs using SPSS version 22 (IBM Corporation, New York, USA). Data were expressed as mean ± SD. The Shapiro–Wilk test was used to check the normality of data. To test the assumption of homogeneity of variance, Levin's test was used. One-way analysis of variance (ANOVA) was used to analyse the difference in means of zones of inhibition, minimum inhibitory concentration, and minimum bactericidal concentration using SPSS version 22. To find out whether there was a significant difference between the groups, a Bonferroni post hoc test was used. Differences were considered statistically significant at *p* < 0.05.

## 3. Results and Discussion

Studies conducted in several countries have reported that the use of compounds extracted from medicinal plants may be beneficial in the development of antibiotics [34]. However, the antibacterial activity studies of the widely used medicinal plants, including *P. tinctorius,* are limited [22]. The current study evaluated the antibacterial activity of *P. tinctorius* against *Escherichia coli*, *Salmonella typhi*, and *Shigella dysenteriae* (Figure 1). In the current study, the greatest susceptibility of the methanolic subextract (M1) was exhibited by *Shigella dysenteriae*, followed by *Salmonella typhi*, and the least was *Escherichia coli* at both 200 mg/mL and 100 mg/mL (Tables 1 and 2). This could be attributed to the different types of antigens present in these bacteria. The three bacteria, *Shigella dysenteriae*, *Salmonella typhi*, and *Escherichia coli*, have O-antigens, which have been reported to be different even within the bacteria genera of *Salmonella* [35, 36] and *Shigella* [37]. However, besides O-antigen, *Salmonella typhi* contains K-antigen, while *Escherichia* has K-antigen and H-antigen [35]. The presence of extra antigens on *Escherichia coli* could account for the amplified stimulation of immune response observed as having been greatest for *Escherichia coli* followed by *Salmonella typhi* and least for *Shigella dysenteriae*, when exposed to the 100 mg/mL and 200 mg/mL methanolic subextract. This response may have been protective of *Escherichia coli* against the methanolic subextract and rendering *Escherichia coli* less susceptible than *Shigella dysenteriae* and *Salmonella typhi*. Antigens have been reported to stimulate or activate the immune system of bacteria and protect them from the bactericidal effects of drugs [38]. Furthermore, the cell wall peptidoglycan layer covering Gram-negative bacteria is highly cross-linked in *Escherichia coli* than in *Shigella dysenteriae* and *Salmonella typhi* [39]. This partially prevented entering of the active compounds into *Escherichia coli*, thus rendering *Shigella dysenteriae* and *Salmonella typhi* more susceptible to the extract than *Escherichia coli*. Chloroform subextract was only active on *Shigella dysenteriae* at 200 mg/mL and 100 mg/mL. However, the mean zones of inhibition of the chloroform subextract were less than those of the methanolic subextract at 100 mg/mL and 200 mg/mL. This suggests that the lipopolysaccharide outer membrane of *Shigella dysenteriae* is more permeable to the active constituents in chloroform subextract than *Salmonella typhi* and *Escherichia coli*, and this could result in higher inhibition of metabolism processes for *Shigella dysenteriae* than for *Salmonella typhi* and *Escherichia coli.* The hexane subextract did not show antibacterial activity for all tested bacteria at 100 mg/mL and 200 mg/mL. This implies that the nonpolar compounds such as oils, fats, and fatty acids extracted by hexane from the crude methanolic extract (M) have no antibacterial activity against *Escherichia coli, Shigella dysenteriae*, and *Salmonella typhi*. It can therefore be said that of the three extracts tested, the methanol subextract was the only extract that showed activity against *Escherichia coli, Shigella dysenteriae*, and *Salmonella typhi* at 200 mg/mL and 100 mg/mL. This antibacterial activity could be attributed to the polar compounds present in the methanolic subextract [40]. In the genus *Pterocarpus*, it is reported that *P. angolensis* stem bark chloroform and hexane subextracts showed no activity against *Escherichia coli*, *Pseudomonas aeruginosa*, and *Staphylococcus aureus* except for the weak response of the chloroform extract against *Bacillus subtilis* [41]. Methanolic extract of *Pterocarpus indicus* has also been reported to possess higher antibacterial activity compared to ethyl acetate extracts [42].

Generally, there was less activity of the methanolic subextract of *P. tinctorius* against *Escherichia coli, Shigella dysenteriae*, and *Salmonella typhi* compared to the standard drugs, namely, ciprofloxacin and nalidixic acid (Tables 1 and 2). This may be attributed to the fact that the methanolic subextract contains small amounts of active compounds. This further suggests that some of the compounds found in the methanolic subextract (M1) may have caused an antagonistic effect on the active compounds that possess antibacterial activity against tested bacteria [43]. However, ciprofloxacin and nalidixic acid are pure compounds that have no other compounds that can cause antagonistic effects to the active compound. Ciprofloxacin and nalidixic acid are quinolone types of antibiotics and their mechanism of action is to inhibit DNA gyrase, an enzyme specific and essential for all bacteria. As a result, DNA synthesis is inhibited [12]. Perhaps, some of the active constituents found in the bark of *P. tinctorius* might have the same mechanism of action as quinolones against the tested bacteria. In the current study, the concentration of the extract also affected the antibacterial activity. It was observed that the higher the concentration of the methanolic subextract used for antibacterial testing, the higher the antibacterial activity; thus, the activity was dose-dependent.

Phytochemical analysis of subfraction FM2 was performed using qualitative phytochemical methods. The findings of this study show that the active ingredients that may be responsible for the antibacterial activity of subfraction FM2 could be phenolics, flavonoids, tannins, and saponins (Table 3). The genus *Pterocarpus* is rich in phenolics. It is reported that tannins, flavonoids, saponins, and phenols in the genus *Pterocarpus* (*Pterocarpus erinaceus*, *Pterocarpus marsupium*, *P. angolensis, P. osum, P. soyauxii*, and *P. santalinoides*) possess antibacterial activities against *Escherichia coli*, *Staphylococcus aureus* [19, 27, 44, 45], *Salmonella typhi, Bacillus subtilis*, *Pseudomonas aeruginosa* [20, 46], and *Shigella flexneri* [47].

In the current study, subfractions FM1 and FM3 did not exhibit any antibacterial activity. This suggests that compounds responsible for inhibiting tested bacteria did not spread across subfractions FM1 and FM3. The higher activity of *Shigella dysenteriae* was statistically significant (*p* < 0.05), when compared to *Escherichia coli* and *Salmonella typhi* (Table 4). This possibly suggests that the hydrophobic pathways found on the outer membrane of *Shigella dysenteriae* are more permeable to active compounds found in subfraction FM2 compared to those of *Escherichia coli* and *Salmonella typhi* [37, 48]. The mean zone of inhibition for *Salmonella typhi* was higher than *Escherichia coli* for subfraction FM2 at 10 mg/mL but not statistically significant (*p* < 0.05). This could be attributed to the genetic differences between these two strains [49].

The antibacterial activity of the methanolic subextract (M1) showed less antibacterial activity at 200 mg/mL on *Shigella dysenteriae* compared to the purified subfraction FM2, with the highest zone for *Shigella dysenteriae.* This is because FM2 is a more isolated fraction with fewer specific groups of compounds that are active against *Shigella dysenteriae*, *Salmonella typhi*, and *Escherichia coli*. Therefore, purification of the methanolic extract increased the concentration and antibacterial activity of the extract against the tested micro-organisms [49]. This finding is in line with [45], who reported that the methanol fraction of *Pterocarpus erinaceus* was found to be more active than the crude methanolic extract with zones of 20 mm and 30 mm against *Escherichia coli*, respectively.

Gas chromatography and mass spectrometry (GC-MS) analysis of ethanol and water, ethyl acetate, and benzene-ethanol heartwood extracts of *P. tinctorius* showed the presence of pterostilbene, dibutyl phthalate, 4-tert-butyl-2-[4-nitrophenyl] phenol, 4H-1-benzopyran-4-one, and 5-hydroxy-2-(4-hydroxyphenyl)-7-methoxy [21]. Pterostilbene has been isolated from the members of the genus *Pterocarpus* such as *P. santalinus, P. marsupium,* and *P. soyauxii* [50, 51] and has been reported to exhibit antibacterial activity against *Escherichia coli* and *Klebsiella pneumonia* [52]. Reference [21] reported a higher content of pterostilbene in the heartwood of *P. tinctorius* (99.2%) than in *P. santalinus* (16.5%). Perhaps, the antibacterial activities observed in our study could be attributed to some of these compounds isolated from *P. tinctorius*.

In this study, *S. dysenteriae* had the lowest MIC compared to *S. typhi* and *E. coli* (Table 5) This could be attributed to the reduction of the therapeutic concentration of the active constituents in *Escherichia coli* than *Salmonella typhi* due to the higher biochemical activity of *Escherichia coli* [53].The difference in MIC from 0.62 mg/mL for *Shigella dysenteriae* to 1.25 mg/mL for *Salmonella typhi* and *Escherichia coli* is statistically significant at *p* < 0.05. This could be explained by the difference in the structure of the lipopolysaccharide found on the cell surface of the tested bacteria [54]. This lipopolysaccharide is thicker for *Escherichia coli* and *Salmonella typhi* than for *Shigella dysenteriae*[48]. Hence, it provided a barrier that could not easily allow penetration of active compounds into *Salmonella typhi* and *Escherichia coli* but could easily penetrate the thinner structure of *Shigella dysenteriae.* This resulted in a higher susceptibility of *Shigella dysenteriae* than *Salmonella typhi* and *Escherichia coli* to fraction FM2. In the genus *Pterocarpus*, friedelin has been isolated and identified using HPLC and NMR from methanolic and dichloromethane of *Pterocarpus erinaceus* [55]. Reference [56] reported an in vitro antibacterial activity of friedelin isolated from ethyl acetate leaf extract of *Pterocarpus santalinoides* using the broth microdilution method. Friedelin has been reported to have antibacterial activity against methicillin-resistant*Staphylococcus aureus* (MRSA), *Staphylococcus aureus*, *Streptococcus pneumonia*, *Campylobacter jejuni*, *Helicobacter pylori*, and *Escherichia coli* with an MIC ranging 2.5–20 *μ*g/mL [56]. Reference [57] has characterised methanolic extracts of *Pterocarpus erinaceus* such as friedelin, 2, 3-dihydroxypropyl octacosanoate, *β*-sitosteryl-*β*-D-glucopyranoside, and a mixture of *β*-sitosterol, stigmasterol, and campesterol, which showed antibacterial activity against methicillin-resistant*Staphylococcus aureus* and *Pseudomonas aeruginosa* with MIC values ranging from 32 *µ*g/mL to 256 *µ*g/mL. In this study, it is presumed that possibly these compounds, which have been isolated from the genus *Pterocarpus*, could also be responsible for the antibacterial activity of subfraction FM2.

According to the MIC standard guidelines provided by [58, 59], plant extracts that have a MIC value of between 0.5 mg/mL and 1.6 mg/mL are considered to be moderate inhibition, while a MIC value of less or equal to 0.5 mg/mL has strong antibacterial activity, and a MIC of greater or equal to 1.6 mg/mL is considered weak. Therefore, *Shigella dysenteriae* with a MIC value of 0.62 mg/mL, *Salmonella typhi*, and *Escherichia coli* with an MIC value of 1.25 mg/mL were moderately inhibited by subfraction FM2. However, subfraction FM2 was not a pure compound. Therefore, low values of MIC could be obtained upon purification of subfraction FM2.

There was no growth of bacteria observed on all three plates after subculturing of bacteria for 24 hours. The results indicate that the active ingredients found in subfraction FM2 of *P. tinctorius* have bactericidal effects on *Shigella dysenteriae*, *Salmonella typhi*, and *Escherichia coli*. In the genus *Pterocarpus*, MBC of *Pterocarpus lucens* at 50 mg/mL against *Escherichia coli*, *Shigella dysenteriae*, and *Salmonella typhi* has been reported [60]. The equality in MIC and MBC suggests that subfraction FM2 has active constituents that can inhibit and kill the growth of *Escherichia coli*, *Shigella dysenteriae*, and *Salmonella typhi* at 0.62 mg/mL and 1.25 mg/mL, respectively. In the genus *Pterocarpus*, polyphenols such as (-)-epicatechin, epicatechin-3-O-galate, epicatechin (4b–8)-epicatechin (B2), and a hexamer of epicatechin have been isolated from ethanolic bark extract of *P. angolensis* and *P. marsupium*, using nuclear magnetic resonance (NMR), gas chromatography-mass spectrometry (GC–MS), and high-performance liquid chromatography (HPLC) [51]. These compounds have been found to have antibacterial activity with an MBC of <0.5 mg/mL against *Staphylococcus aureus*, *Salmonella typhi*, *Micrococcus kristinae*, and *Acinetobacter calcoaceticus* [61]. Likewise, separation and characterisation of methanolic extract of *P. indicus* using GC and MS showed the presence of a sapogenin (campesterol), cyclopropane, and 2,6-bis (1,1-dimethylethyl)-4-methyl as compounds with bactericidal activity against *Escherichia coli, Staphylococcus aureus,* and *Bacillus subtilis* [62]. Perhaps, some of these isolated compounds from the genus *Pterocarpus* may be responsible for the bactericidal effects observed in our study. This is because the chemistry of the genus of the plant under investigation could provide hints regarding the possible chemical compound or compounds responsible for specific medicinal activity [24, 63].

Bactericidal effects of subfraction FM2 further imply that the active compounds in fraction FM2 targeted porins and rendered bacteria more permeable to the active compounds. Porins are the major outer membrane protein structures found in Gram-negative bacteria, and they control the permeability of the bacteria membrane [12]. Porins have been reported to be the potential targets for the bactericidal effects of drugs [64]. The empiric antibiotic therapy in adults for the treatment of gastroenteritis is fluoroquinolone such as ciprofloxacin and nalidixic acid [65]. However, fluoroquinolones have been reported to have multiple side effects such as tendonitis, tendon rupture, arthralgia, pain in extremities, gait disturbance, neuropathies associated with paraesthesia, depression, fatigue, memory impairment, sleep disorders, and impaired hearing, vision, taste, and smell [11, 51]. Therefore, subfraction FM2 could be isolated into compounds that may be free from these side effects.

## 4. Conclusion

The study confirms that methanolic subextract (M1) and its subsequent methanolic subfraction FM2 of *Pterocarpus tinctorius* possess antibacterial activity against *Shigella dysenteriae*, *Salmonella typhi*, and *Escherichia coli*. However, the chloroform subextract only exhibited antibacterial activity against *Shigella dysenteriae,* while hexane subextract had no activity against any tested bacteria. Qualitative phytochemical analysis of subfraction FM2 revealed phenolics, flavonoids, tannins, and saponins as active constituents which could be responsible for the antibacterial activity of the methanolic bark extract of *Pterocarpus tinctorius*. Further studies to purify and perform structural elucidation of the phytochemicals responsible for antibacterial activity are strongly recommended. The results provide a scientific basis for the traditional use of the bark of *Pterocarpus tinctorius* in the treatment of bacterial gastroenteritis. The findings of this study thus corroborate the use of *Pterocarpus tinctorius* in the treatment of bacterial gastroenteritis in traditional medicine.

## Figures and Tables

**Table 1:** Antibacterial activity of stem bark subextracts of *P. tinctorius* against a standard strain and clinical isolates at 100 mg/mL.

			Zones of inhibition		
*Micro-organism*	Methanol	Chloroform	Hexane	Nalidixic acid	Ciprofloxacin
*Escherichia coli* (ATCC 25923)	8.3 ± 0.1^*∗*^	NZ	NZ	17.9 ± 0.1	24.2 ± 0.2
*Salmonella typhi*	11.9 ± 0.3^*∗*^	NZ	NZ	17 ± 0.2	24.1 ± 0.1
*Shigella dysenteriae*	12.3 ± 0.1	10 ± 0.1	NZ	17.0 ± 0.1	24.2 ± 0.1

Zones of inhibition in millimetres after excluding 6 mm well diameter for 100 mg/mL of the subextracts of methanol, chloroform, and hexane; 5 *µ*g/disk for ciprofloxacin and 30 *µ*g/disk for nalidixic acid. NZ, no zone of inhibition was observed. All values are expressed as mean ± SD (*n* = 3). Values with superscript ∗ differ significantly from *Shigella dysenteriae* values at *p* < 0.05. ^*∗*^*p* < 0.001.

**Table 2:** Antibacterial activity of stem bark subextracts of *P. tinctorius* against a standard strain and clinical isolates at 200 mg/mL.

Zones of inhibition					
Micro-organism	Methanol	Chloroform	Hexane	Nalidixic acid	Ciprofloxacin
*Escherichia coli* (ATCC 25923)	12.2 ± 0.1^*∗*^	NZ	NZ	17.8 ± 0.0	23.8 ± 0.1
*Salmonella typhi*	13.7 ± 0.3^*∗*^	NZ	NZ	17.1 ± 0.1	24.0 ± 0.0
*Shigella dysenteriae*	14.3 ± 0.2	11.2 ± 0.0	NZ	17.3 ± 0.1	24.2 ± 0.1

Zones of inhibition in millimetres after excluding 6 mm well diameter for 200 mg/mL of the subextracts of methanol, chloroform, and hexane; 5 *μ*g/disk for ciprofloxacin and 30 *μ*g/disk for nalidixic acid. NZ, no zone of inhibition was observed. All values are expressed as mean ± SD (*n* = 3). Values with superscript ^*∗*^ differ significantly from *Shigella dysenteriae* values at *p* < 0.05. ^*∗*^*p* < 0.001.

**Table 3:** Antibacterial activity of subfractions against three bacterial strains at 10 mg/mL.

Fraction	Zone of inhibition		
	*Shigella dysenteriae*	*Salmonella typhi*	*Escherichia coli* (ATCC 25923)
FM1	NZ	NZ	NZ
FM2	20 ± 0.1	18.0 ± 0.1^*∗*^	17.2 ± 0.3^*∗*^
FM3	NZ	NZ	NZ
Ciprofloxacin	21 ± 0.1	20 ± 0.0	20 ± 0.2

Zones of inhibition in millimetres after excluding 6 mm of well diameter for 10 mg/mL of the subfractions and 0.01 mg/mL for ciprofloxacin. NZ, no zone of inhibition was observed. All values are expressed as the mean (*n* = 3). Values with superscript ∗ differ significantly from *Shigella dysenteriae* values at *p* < 0.05. ^*∗*^*p* < 0.001.

**Table 4:** Minimum inhibitory concentration and minimum bactericidal concentration of active subfraction FM2 and ciprofloxacin against three bacterial strains.

Fraction	Minimum inhibitory concentration (mg/mL) and minimum bactericidal concentration					
	*Shigella dysenteriae*		*Salmonella typhi*		*Escherichia coli* (ATCC 25923)	
	MIC	MBC	MIC.	MBC	MIC	MBC
FM2	0.62 ± 00	0.62 ± 00	1.25 ± 00^∗^	1.25 ± 00^∗^	1.25 ± 00^∗^	1.25 ± 00^∗^
Ciprofloxacin	2 × 10^−4^		7 × 10^−4^		7 × 10^−4^	

All values are expressed as the mean (*n* = 3). Values with superscript ∗ differ significantly from *Shigella dysenteriae* values at *p* < 0.05. ^*∗*^*p* < 0.001.

**Table 5:** Phytochemicals present in active subfraction FM2 of *P. tinctorius*.

S. no.	Phytochemicals	Type of test	Present/absent
1	Alkaloids	Dragendorff's	−
2	Phenolics	Ferric chloride	+++
3	Tannins	Ferric chloride	+++
4	Flavonoids	Alkaline reagent	++
5	Saponins	Froth test	+

+++ = intense, ++ = intermediate, + = low, and − = absent.

## Data Availability

The data used to support the findings of this study are available from the corresponding author upon request.
